# Exploring how attachment insecurities mediate the link between sexual and partnership satisfaction in adolescents and young adults with cancer

**DOI:** 10.2340/1651-226X.2025.42110

**Published:** 2025-01-21

**Authors:** Svenja Heyne, Hannah Brock, Diana Richter, Annekathrin Sender, Jenny Rosendahl, Michael Friedrich, Kristina Geue, Anja Mehnert-Theuerkauf

**Affiliations:** a Department of Medical Psychology and Medical Sociology, University Medical Center Leipzig, Leipzig, Germany; b Comprehensive Cancer Center Central Germany (CCCG), Leipzig, Germany; c Institute of Psychosocial Medicine, Psychotherapy and Psychooncology, Jena University Hospital, Jena, Germany; d University Clinic for Psychosomatic Medicine and Psychotherapy, University Medical Center Magdeburg, Magdeburg, Germany

**Keywords:** Sexual health, attachment-related anxiety, attachment-related avoidance, AYA cancer survivors, oncology, supportive care

## Abstract

**Background and purpose:**

Comprehensive data on factors affecting partnership satisfaction among adolescents and young adult (AYA) cancer survivors are limited. Our study examines partnership satisfaction, sexual satisfaction, and attachment insecurities, exploring how attachment-related anxiety and avoidance influence the relationship between sexual and partnership satisfaction across major tumor entities in this population.

**Patients and methods:**

We utilized data from two measurement time points (t1 and t6) of the AYA-LE study, a prospective longitudinal investigation examining the temporal course and associated factors of life satisfaction and psychological distress among AYA cancer survivors. We examined the mediating effect of attachment insecurities (ECR-RD) on the relationship between sexual satisfaction (FLZ-Sex) and partnership satisfaction (PFB), while controlling for sociodemographic and clinical characteristics, in a sample of *N* = 275 participants.

**Results:**

Higher sexual satisfaction was correlated with lower attachment-related anxiety (*r* = -0.51, *p* < 0.001) and lower attachment-related avoidance (*r* = -0.49, *p* < 0.001). Both lower attachment-related anxiety and attachment-related avoidance were correlated with higher partnership satisfaction (*r* = -0.64, *p* < 0.001 and *r* = -0.72, *p* < 0.001, respectively). Sexual satisfaction partially predicted partnership satisfaction of AYA cancer survivors through attachment-related anxiety and attachment-related avoidance while the mediating effect accounted for 75% of the total effect.

**Interpretation:**

The associations between sexual satisfaction, partnership satisfaction, and attachment highlight the need to address emotional and relational aspects in supportive care for AYA cancer survivors. There is a clear need for more targeted studies on attachment patterns, sexual satisfaction, and partnership satisfaction in this specific population to further refine and validate these approaches.

## Introduction

Adolescents and young adults (AYAs), aged 15 to 39 years at diagnosis, represent a unique group of cancer survivors facing distinct psychosocial and sexual health challenges [1, 2]. AYA cancer survivors experience several life events, including physical maturation, formation of romantic partnerships, and exploration of intimacy during a critical developmental period [3, 4]. Cancer and its treatment can interfere with or postpone these life experiences, thereby affecting the psychosexual well-being of AYA cancer survivors [1].

Romantic relationships in adulthood are influenced by early attachment styles formed in childhood [5]. Attachment anxiety (fear of abandonment) and attachment avoidance (defensive independence) are two factors underlying the concept of attachment insecurity (AI) [6]. Attachment insecurity, particularly avoidance, is linked to diminished sexual and partnership satisfaction (PSAT), likely stemming from negative thoughts and distress about sexual encounters [7–9]. Insecure attachment styles increase distress, hinder emotional intimacy, and create challenges in forming and maintaining satisfying relationships [10–12].

Cancer treatments can lead to long-term sexual dysfunctions, which can further affect self-perception [13]. These disruptions in sexual satisfaction (SSAT) are closely associated with psychological challenges, including depression and decreased self-esteem [14]. For individuals managing chronic illnesses, such as cancer, partnerships play a pivotal role in shaping health outcomes [15]. Emotional support from a partner can reduce isolation, enhance coping, and improve mental health [16] by alleviating psychological distress and fostering emotional resilience in this population [17, 18].

It is essential to understand the complex dynamics that contribute to PSAT within this population. For this young population, navigating the challenges of cancer during a critical developmental period, the interplay between attachment style and partnership outcomes may have profound implications for their overall well-being and quality of life.

Therefore, this study aims to (1) investigate the levels of PSAT, SSAT, and AI across sociodemographic and medical factors and (2) explore how AI, i.e. attachment-related anxiety (AAX) and attachment-related avoidance (AAV) influence the relationship between SSAT and PSAT among AYA cancer survivors across major tumor entities. By addressing these aims, this research enhances our understanding of the psychosocial needs of AYA cancer survivors, emphasizing the importance of encompassing not only medical aspects but also emotional and social factors.

## Patients and methods

### Study design and sample

We used data from the first and last measurement time point (t1: 05/2014-12/2015 and t6: 05/2021-09/2021) of the AYA-LE study, a prospective longitudinal study with six measurement time points investigating the temporal course and related factors of life satisfaction and psychological distress of AYA cancer survivors. The t6 survey focused specifically on AYA social relationships, such as partnership and sexuality [19, 20].

Patients were eligible for participating in the study if they (1) were between 18 and 39 years of age at diagnosis, (2) had first diagnosis of a cancer at any tumor site (C00–C97), (3) were diagnosed within the last 4 years at t1, and (4) were able to speak German and physically and cognitively able to participate. All participants gave written informed consent in accordance with the Declaration of Helsinki. The study was approved by the Research Ethics Committee of the University of Leipzig (Ref. 372–13–16,122,013).

### Study recruitment and data collection

The total recruitment process in the AYE-LE study ran for a period of 88 months (from 05/14 to 09/21) in cooperation with 16 oncological acute care hospitals, two local tumor registries, and four (cancer) rehabilitation clinics specialized in treating AYA cancer survivors [21]. Patient recruitment in t6 was carried out from May 2021 to September 2021.

Patients who consented to participate received participant documents and the questionnaire by mail or could complete it online using the software *LimeSurvey.* Reminders were sent continuously every 10 days via email. A first postal reminder was sent after 4 weeks and a second postal reminder 3 weeks later.

### Study measures

#### Sociodemographic and clinical characteristics

Sociodemographic characteristics, including sex at t1, age, partnership duration, and housing situation at t6, were obtained from patients’ self-reports. Clinical characteristics, including cancer diagnosis as reported by patients at t1, along with any new cancer-related complications due to cancer and its treatment, cancer recurrence, metastases, or second cancer diagnoses reported at t6 since t5-survey, were documented based on patient self-reports.

#### Sexual satisfaction

SSAT was assessed using the validated life satisfaction questionnaire (FLZ) – sexuality scale (FLZ-Sex) [22]. The FLZ-Sex uses seven items to quantify SSAT considering physical attraction, sexual efficiency, sexual contacts, sexual response, sexual partner interaction, communication, and sexual reactions. Participants are supposed to rate on a seven-point Likert-Scale from ‘very unsatisfied’ (1) to ‘very satisfied’ (7). The sum score of the scale ranges from 7 to 49 with higher scores indicating a higher level of SSAT [22]. The questionnaire demonstrates strong internal consistency, as indicated by a Cronbach’s alpha of 0.92 [23].

#### Partnership satisfaction

PSAT was assessed using the marital quality questionnaire (PFB) [24]. The PFB uses 30 items to measure marital quality on three subscales, that is tenderness (T), quarreling (Q), and togetherness/communication (TC). Each subscale contains 10 items with a four-point Likert scale asking participants to indicate whether their partner ‘never/almost never’ (0) to ‘quite often’ (3) exhibits a particular behavior. The overall quality of relationship score can be obtained with the following equation: PFB = (30 – Q) + T + TC and ranges from 0 to 90 with higher scores indicating a higher level of PSAT [24]. The questionnaire demonstrates strong internal consistency, as indicated by a Cronbach’s alpha of 0.94 [24].

#### Attachment insecurities

AI in adults was assessed using the validated German revised short version of the Experience of Close Relationships (ECR-RD8) [25]. The ECR-RD8 captures attachment-related cognitions and expectations with regard to romantic relationships on two scales: AAX and AAV. In the short version, each scale comprises four items to be rated on a 7-point Likert scale from ‘strongly disagree’ (1) to ‘strongly agree’ (7). Scores ranged from 8 to 56 with higher scores on one or both scales indicate an insecure romantic attachment style, whereas lower scores suggest a secure attachment style [25]. The questionnaire demonstrates good internal consistency, as indicated by a McDonald’s omega > 0,8 [25].

### Statistical analysis

We applied descriptive analyses for both continuous (means, standard deviations) and categorical variables (frequencies, percentages). All four items of the avoidance subscale from the ECR were inverse coded before mean values were computed.

To examine differences in SSAT, PSAT, and AI scores between sociodemographic and clinical characteristics, t-tests or one-way analysis of variance (ANOVA) were calculated. Comparisons between participants and nonresponders were conducted using ANOVA with Bonferroni correction due to multiple comparisons (adjusted α level 0.00625). Linear correlations between two variables were examined with bivariate correlation using Pearson’s *r*.

We conducted parallel mediation using PROCESS model 4 with AAX (M1) and AAV (M2) as mediators and statistically significant covariates from univariate analyses (*p* < 0.05). Mediating effects were estimated through linear regression, following Baron and Kenny’s method [26]. First, SSAT significantly affects AAX and AAV (paths a_1_ and a_2_); second, SSAT significantly influences PSAT (path c); and third, AAX and AAV significantly affects PSAT (paths b_1_ and b_2_). If these conditions hold in the predicted direction, the effect of SSAT on PSAT in the third equation (path c’) was expected to be smaller than in the second (path c), indicating partial mediation if significant. We set bootstrap samples to 10,000 and the significance level to 0.10 for a two-sided test. The proportion mediated was calculated by dividing the indirect effect by the total effect. To test for differences in indirect effects, pairwise contrasts were conducted, with statistical significance determined by whether the confidence interval for the contrast value (C1) did not contain zero. This would indicate that the mediators have a different impact on our dependent variable PSAT.

Data analyses were performed with IBM SPSS Statistics 27 [27] and PROCESS Makro v4.2 for SPSS [28].

## Results

### Sample

**Figure 1:**
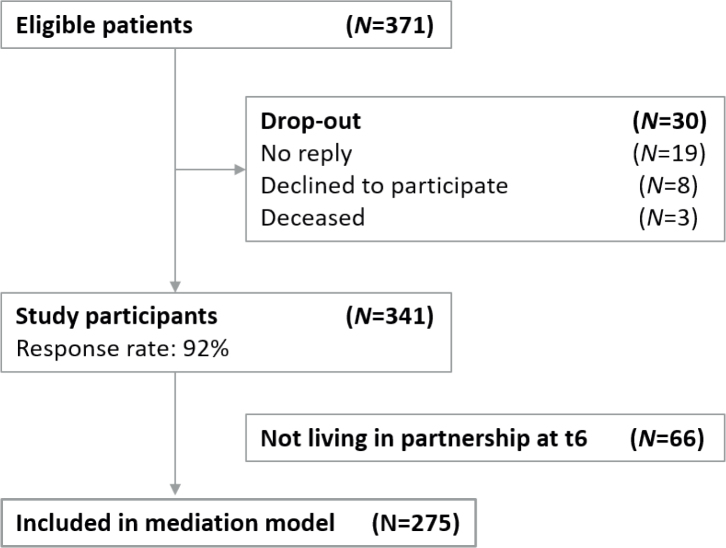
Flowchart of participants. *Notes. N* = sample size.

Of the 371 eligible patients from the fifth measurement time point (t5), 341 (response rate = 91.9%) participated in the study at t6. Of these, 275 participants were in a partnership, had completed the PFB questionnaire, and were therefore included in the final analysis (Figure 1).

#### Nonresponder analysis

Study participants (*N* = 341) at t6 were older (M = 37.10 years; SD = 6.11, *p* ≤ 0.001) differed in cancer entity (*p* = 0.012) with a higher percentage of breast cancer (28.7% vs. 9.8%) and a lower percentage of hematological cancers (6.0% vs. 14.6%) compared to nonresponders (*N* = 30). There were no significant differences in sex between both groups (*p* = 0.262).

### Sample characteristics and scores of SSAT, AAX, AAV, and PSAT

Sample characteristics and differences in mean scores on the SSAT, PSAT, and both AAX and AAV across sociodemographic and clinical characteristics for participants who were in a partnership and were thus included in the final analysis are presented in Table 1. Participants had a mean age of 36.82 ± 6.23 years and an average partnership duration of 10.39 ± 7.10 years. For SSAT, the mean score on the FLZ-Sex was 31.95 ± 10.03. The mean scores on the ECR-RD for AAX and AAV were 2.26 ± 1.42 and 2.10 ± 1.27, respectively. Regarding PSAT, the mean score on the PFB total scale was 66.33 ± 16.02. For the subscales, the mean scores were 5.75 ± 5.40 for Q, 21.93 ± 6.17 for TC, and 20.18 ± 6.82 for T.

**Table 1:** Sample characteristics from t6 participants that were in a partnership and differences in means among SSAT, PSAT, AAX, and AAV.

	Total sample	SSAT	PSAT^2^	AI	
				AAX	AAV
	*n* (%)	M ± SD	M ± SD	M ± SD	
	265 (100)				
**Sociodemographic data**					
Sex					
Male	58 (21.9)	34.34 ± 8.98	63.82 ± 15.33	2.08 ± 1.32	2.32 ± 1.37
Female	207 (78.1)	31.28 ± 10.23	67.03 ± 16.31	2.31 ± 1.44	2.03 ± 1.24
*T*		2.068	-1.349	-1.074	1.533
*p*		0.040*	0.178	0.284	0.126
*d*		0.31	-0.20	-0.16	0.22
Age [in yrs]					
≤ 25	12 (4.5)	34.33 ± 8.03	71.12 ± 12.71	2.06 ± 1.20	2.01 ± 1.29
26–30	62 (23.4)	33.48 ± 9.10	67.42 ± 15.38	2.23 ± 1.59	1.98 ± 1.11
31–35	61 (23.0)	31.21 ± 9.69	65.21 ± 14.56	2.30 ± 1.28	2.09 ± 1.07
36–40	57 (21.5)	29.10 ± 11.98	62.58 ± 18.83	2.37 ± 1.53	2.24 ± 1.53
> 41	73 (27.5)	32.83 ± 8.15	61.96 ± 19.47	2.79 ± 1.36	2.50 ± 1.10
*F*		2.938	2.737	0.643	0.592
*p*		0.021*	0.029*	0.632	0.669
*Eta^2^*		0.04	0.04	0.01	0.01
Partnership duration [in yrs]^a^					
< 5	61 (23.0)	34.70 ± 9.23	68.83 ± 15.61	2.43 ± 1.60	2.04 ± 1.23
5–9	47 (17.7)	32.55 ± 9.29	70.14 ± 13.49	1.84 ± 1.20	1.82 ± 0.99
10–14	46 (17.4)	32.58 ± 9.74	68.06 ± 12.81	2.34 ± 1.50	1.94 ± 1.01
15–19	31 (11.7)	28.58 ± 11.18	61.20 ± 18.35	2.39 ± 1.39	2.66 ± 1.56
> 20	30 (11.3)	29.26 ± 10.80	60.52 ± 16.95	2.13 ± 1.21	2.02 ± 1.18
*F*		2.712	3.224	1.405	2.454
*p*		0.031*	0.014*	0.233	0.041*
*Eta^2^*		0.04	0.05	0.02	0.04
Housing situation					
Alone	27 (10.2)	32.25 ± 10.42	62.87 ± 21.98	3.07 ± 1.65	2.67 ± 1.47
Cohabiting with a partner	232 (87.5)	31.84 ± 10.05	66.55 ± 15.34	2.17 ± 1.36	2.04 ± 1.24
Other	6 (2.3)	34.83 ± 8.58	73.33 ± 6.95	2.20 ± 1.48	1.54 ± 0.71
*T*		0.273	1.226	5.047	3.577
*p*		0.762	0.295	0.007**	0.029*
*Eta^2^*		0.01	0.01	0.03	0.02
**Clinical data**					
Cancer Diagnosis					
Breast	76 (28.7)	29.02 ± 11.10	63.93 ± 17.04	2.38 ± 1.45	2.14 ± 1.27
Gynaecological	20 (7.5)	31.15 ± 11.78	68.67 ± 16.79	2.62 ± 1.43	2.08 ± 1.29
Testicular	22 (8.3)	32.31 ± 8.30	59.62 ± 16.60	2.22 ± 1.51	2.27 ± 1.07
Thyroid	17 (6.4)	35.29 ± 6.77	74.16 ± 13.41	1.64 ± 0.97	1.55 ± 0.85
Hematological	92 (34.7)	33.57 ± 8.84	66.33 ± 16.15	2.10 ± 1.29	2.17 ± 1.37
Sarcoma	8 (3.0)	31.12 ± 11.31	67.49 ± 14.72	2.40 ± 1.85	1.93 ± 1.38
Gastrointestinal	7 (6.5)	31.71 ± 14.07	65.29 ± 21.01	2.50 ± 1.68	2.03 ± 1.61
Other	23 (8.7)	33.34 ± 9.94	64.11 ± 17.78	2.52 ± 1.80	1.98 ± 1.20
*F*		1.658	1.781	1.032	0.591
*p*		0.120	0.091	0.409	0.763
*Eta^2^*		0.04	0.05	0.02	0.01
Cancer recurrence					
No	257 (97.0)	31.83 ± 10.10	66.12 ± 16.19	2.26 ± 1.42	2.12 ± 1.28
Yes	8 (3.0)	35.62 ± 7.34	73.13 ± 6.64	2.18 ± 1.29	1.43 ± 0.53
*T*		-1.051	-1.219	0.153	1.496
*p*		0.294	0.224	0.879	0.136
*Cohens’s d*		-0.37	-0.43	0.05	0.53
Metastases					
No	257 (97.0)	32.14 ± 10.01	66.63 ± 15.90	2.26 ± 1.42	2.08 ± 1.26
Yes	8 (3.0)	25.62 ± 9.33	56.57 ± 17.98	2.34 ± 1.23	2.50 ± 1.76
*T*		1.818	1.756	-0.162	-0.897
*p*		0.070	0.080	0.871	0.370
*d*		0.65	0.62	-0.05	-0.32
Cancer-related complications^1^					
No	228 (86.0)	32.65 ± 9.82	67.08 ± 15.63	2.21 ± 1.35	2.05 ± 1.23
Yes	35 (13.2)	26.88 ± 10.19	60.66 ± 17.75	2.59 ± 1.77	2.42 ± 1.50
*T*		3.218	2.222	-1.478	-1.603
*p*		<0.001***	0.027*	0.141	0.110
*d*		0.58	0.40	-0.26	-0.29
Second cancer diagnosis^b^					
No	261 (98.5)	32.04 ± 10.04	66.32 ± 16.13	2.27 ± 1.42	2.09 ± 1.28
Yes	4 (1.5)	25.50 ± 8.58	66.67 ± 6.94	1.25 ± 0.35	2.25 ± 0.88
*T*		1.297	-0.043	1.440	-0.235
*p*		0.196	0.966	0.151	0.815
*d*		0.65	-0.02	0.72	-0.11

SSAT: sexual satisfaction; PSAT: partnership satisfaction; AI: attachment insecurities; AAX: attachment-related anxiety; AAV: attachment-related avoidance; yrs: years; n: sub-sample size; M: mean; SD: standard deviation; T: t-value; p: level of statistical significance based on chi-square-tests and t-test; F: f-value;.

a n/a=50,

b n/a=2,

1 most common complications referred to lymphedema, fatigue, hormonal imbalances, and reduced physical capacity,

2 PFB – global scale,

* significant on a level of *p* < 0.05,

** significant on a level of *p* < 0.01,

*** significant on a level of *p* < 0.001.

### The relationship between SSAT, AAX, AAV, and PSAT

SSAT was significantly positive correlated with PSAT (*r* = 0.57, *p* < 0.001, 95%-CI [0.482, 0.646]). SSAT was significantly negative correlated with AAX (*r* = -0.51, *p* < 0.001, 95%-CI [-0.589, -0.433]) and negative correlated with AAV (*r* = -0.49, *p* < 0.001, 95%-CI [-0.566, -0.404]). AAX was significantly negative correlated with PSAT (*r* = -0.64, *p* < 0.001, 95%-CI[-0.708, -0,566]) and AAV was significantly negative correlated with PSAT (*r* = -0.72, *p* < 0.001, 95%-CI[-0.770, -0.652]).

### Mediation role of AAX and AAV on SSAT and PSAT

**Figure 2:**
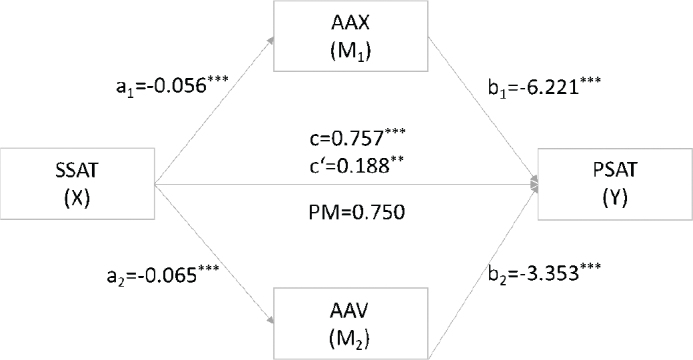
Mediating role of AAX (M1) and AAV (M2) on the relationship between SSAT and PSAT. *Notes.* c’: direct effect of X on Y trough M_1_ and M_2_; c: total effect of X on Y. SSAT: sexual satisfaction; AAX: attachment-related anxiety; AAV: attachment-related avoidance; PSAT: partnership satisfaction; PM: proportion mediated, ratio of natural indirect to total effect, *n* = 275.

Table 2 shows the mediating effects of both AAX (M_1_) and AAV (M_2_) with the summarized coefficients and significance values found in the mediation model. Upon incorporating age, partnership duration and cancer-related complications as covariates in the model, given their significance in prior analyses, the mediation analysis demonstrated that SSAT exerted a significant total effect on PSAT (path c: *B* = 0.757, *p* < 0.001). After entering the mediators in the model, SSAT predicted both mediators AAX (path a_1_: *B* = -0.056, *p* < 0.001) and AAV (path a_2_: *B* = -0.065, *p* < 0.001) significantly. Both AAX (path b_1_: *B* = -6.221, *p* < 0.001) and AAV (path b_2_: *B* = -3.353, *p* < .001) predicted PSAT significantly. SSAT still had a significant effect on PSAT after controlling for both AAX and AAV (path c’: *B* = 0.188, *p* = .006, Figure 2). The relationship between SSAT and PSAT is partially mediated by AAX and AAV, combined indirect effect a_1_b_1_+a_2_b_2_ = 0.568, 95%-CI [0.397, 0.757].

**Table 2:** Summary of the mediating effects of AAX and AAV on the relationship between SSAT and PSAT (*n* = 275).

Type	Effect	*B*	*SE*	*T*	*p*	95% CI^a^	
						LLCI	ULCI
**Indirect effects**	SSAT → AAX → PSAT	0.218	0.053	-	-	0.122	0.331
	SSAT → AAV → PSAT	0.350	0.075	-	-	0.215	0.509
**Components**	SSAT → AAX	-0.056	0.009	-6.080	< 0.001	-0.074	-0.038
	AAX → PSAT	-6.221	0.755	-8.240	< 0.001	-7.709	-4.732
	SSAT → AAV	-0.065	0.011	-5.953	< 0.001	-0.087	-0.044
	AAV → PSAT	-3.353	0.712	-4.709	< 0.001	-4.757	-1.949
**Direct effect**	SSAT → PSAT	0.188	0.092	2.039	0.043	0.006	0.370
**Total effect**	SSAT → PSAT	0.757	0.114	6.622	< 0.001	0.531	0.982

*Notes.* ^a^number of bootstrap samples for percentile bootstrap confidence intervals: 10,000.

B: standardized coefficient; SE: standard error; CI: confidence interval; LLCI: lower bounds; ULCI: upper bounds; *T*: *t*-value; *p*: *p*-value; SSAT: sexuality satisfaction; AAX: attachment-related anxiety; AAV: attachment-related avoidance; PSAT: partnership satisfaction.

The proportion mediated by AAX and AAC was 0.750 (PM = 0.218+0.350/0.757), meaning that 75% of the effect of SSAT on PSAT was explained by AAX and AAV. SSAT exerts approximately equal effects on PSAT on the two mediation pathways, specific indirect effect contrast C1 = -0.131, 95%-CI [-0.042, 0.318].

## Discussion

The purpose of this study was to examine the interrelations of AI on the relationship between SSAT and PSAT among AYA cancer survivors across all major tumor entities. Our findings revealed that higher SSAT was correlated with higher PSAT and lower AAX and AAV. Higher PSAT was correlated with lower AAX and AAV. Our mediation analyses further indicated that SSAT plays a partial predictive role in determining PSAT among AYA cancer survivors. This predictive influence operates through the mediation of AAX and AAV.

In our sample, the mean PSAT score was 66.33, slightly higher than the German normative score of 64.90, based on a sample of 1,114 individuals aged 18 to 50 years. However, subscale scores showed differences: higher conflict behavior (CB: M = 6.18 vs. M = 5.40), lower tenderness (T: M = 18.92 vs. M = 20.10), and comparable TC (TC: M = 20.77 vs. M = 20.10) [29]. The higher overall PSAT score suggests that AYA survivors may develop unique relational strengths, such as greater empathy and deeper connections with others. However, these findings also highlight the complexity of relationships for AYA cancer survivors, where increased conflict can coexist with overall satisfaction. Developmental challenges, such as the desire for autonomy while still depending on caregivers, can lead to tensions and emotional distress, potentially diminishing tenderness. It is important to note that not all conflicts are cancer related, as typical adolescent conflicts may also arise during this time [30].

Age significantly influenced PSAT in our sample. Specifically, younger AYA cancer survivors exhibited significantly higher satisfaction levels in their partnerships as older AYA cancer survivors (over 36 years old). This aligns with a Danish study among 151 AYA cancer survivors aged 15–29 years showing positive relationship changes in younger survivors [31], suggesting that younger survivors may exhibit greater adaptability and positive relationship dynamics. This might be due to by age-related differences in communication styles and support systems as younger adults often emphasize openness and immediacy in addressing emotions and challenges, reflecting generational shifts toward greater emotional expression. Additionally, younger survivors frequently benefit from strong support networks, including family and friends, which can alleviate cancer-related stress and enhance PSAT through emotional and practical support [32]. In contrast, older AYA survivors may face greater challenges if their support systems or communication styles are less responsive to the demands of postdiagnosis life. Furthermore, younger survivors often express a need for improved communication about cancer’s impact on their relationships, as effective communication fosters mutual understanding and support, further strengthening PSAT [33].

Our results further revealed that partnership duration significantly influenced PSAT. AYA cancer survivors in long-term partnerships (over 15 years) reported significantly lower satisfaction. Stress related to cancer treatment and survivorship can lead to decreased satisfaction, particularly in long-term partnerships where expectations diverge from postcancer realities. Communication difficulties and maladaptive coping strategies, such as withdrawal or avoidance, often contribute to relationship strain among survivors, particularly those with unsupportive or overly demanding partners [34–38].

AYA cancer survivors experiencing cancer-related complications exhibited significantly lower satisfaction with their partnerships, as ongoing health issues such as fertility concerns, body image disruptions, and mental health issues might strain AYA cancer survivors’ romantic partnerships. A study conducted in the United States involving 40 childhood cancer survivors revealed both positive and negative impacts of childhood cancer on their romantic partnerships in adulthood. However, the effects on physical intimacy were predominantly negative, with concerns related to fertility, such as feeling less desirable due to the inability to have biological children, as well as physical issues like self-consciousness about scars, hair loss, weight gain, erectile dysfunction, and premature menopause [39].

The mean scores for AAX and AAV in our study were 2.26 and 2.10, respectively. These values are slightly lower than those reported in a German evaluation study involving 1,006 healthy individuals with a mean age of 28.92 years, where the mean AAX and AAV scores were 2.77 and 2.36, respectively [40]. These lower scores may reflect adaptive responses to adversity, as cancer survivors often reevaluate relationships and priorities, potentially leading to stronger bonds with family and friends. This enhanced social support can reduce feelings of anxiety and avoidance in relationships, thereby reducing AI [33, 41].

Our mediation analyses indicated that SSAT plays a partial predictive role in influencing PSAT among AYA cancer survivors. Specifically, a higher level of SSAT corresponded to an elevated level of PSAT. This observation aligns with the outcomes of a Polish study involving 237 cancer-free young adults aged 18–25 years, where SSAT emerged as a primary predictor of PSAT in both sexes [42]. This implies that the connection between SSAT and PSAT is not exclusively driven by health status but represents a fundamental aspect of relationship satisfaction in young adults. SSAT holds a similar significance for AYA cancer survivors as it does for noncancer populations, despite the unique health-related challenges they face. The predictive impact operates via the mediation of AAX and AAV. More precisely, an elevated level of SSAT is associated with a decreased level of AAX and AAV. Reduced AAX and AAV aligns with increased PSAT. Findings from a systematic literature review revealed a consistent association between AI (avoidance and anxiety) and decreased SSAT across various relationship types in noncancer populations [43].

### Study strengths and limitations

Although this study involved a considerably large sample size demonstrating similar age distribution and representation of tumor entities to the broader German AYA population types [21], it is essential to interpret our findings within the context of the following limitations.

As we examined PSAT in a cross-sectional setting, this does not allow interferences on causality and potential changes over time could not be considered, thus further studies on longitudinal effects should be conducted.

In our study, frequency of occurrence estimation was based on self-reports. Sexual health and its vulnerability are an issue prone to stigmatization. It is also possible that self-reported data is biased toward underestimation or is a subject to social acceptability bias. On this topic, however, it should be noted that this problem may be masked by patients in face-to-face interviews, so that the assessment by self-report may provide even more valid data.

### Clinical implications

AYA cancer survivors require age-appropriate and flexible care, as well as informational needs and treatment-related education that foster autonomy for long-term survivorship [44]. The observed associations among SSAT, PSAT, and attachment-related factors underscore the necessity for a comprehensive and nuanced approach in designing and implementing psychosocial support interventions for AYA cancer survivors, given the current absence of tailored interventions.

In order to facilitate the targeted development of effective therapeutic interventions, it is essential to assess attachment styles. This can be achieved through the Adult Attachment Interview (AAI) [45]. Rather than focusing on the degree of security or insecurity in childhood attachments, this assessment is concerned with how adults reflect on their early attachment experiences and how they interpret these experiences within the context of their current relationships. Working through unresolved attachment issues with a trained therapist can lead to healthier attachment patterns. Interventions should therefore primarily focus on promoting secure attachment patterns, as securely attached individuals demonstrate superior emotion regulation capabilities, exhibiting a more balanced approach to both positive and negative affective states [46]. This balanced emotional regulation is crucial for maintaining healthy interpersonal relationships and overall psychological well-being. By fostering secure attachment, interventions may facilitate the development of more effective emotion regulation skills, such as reducing worry and rumination, and enhancing pleasure and satisfaction, potentially leading to improved outcomes in both sexual and relational domains for AYA cancer survivors and their partners [6].

Therapeutic interventions addressing sexual concerns should emphasize sexual emotions, such as pleasure, satisfaction, and related states of anxiety and worry, as well as the relational goals associated with sexual activity, rather than focusing on sexual performance. These interventions should also incorporate the perspectives of partners to enhance relational stability and adapt to changing roles in challenging situations. It is further important to explore factors that may help to fix maladaptive and insecure attachment styles and to nourish attachment security, as this is an important protective factor for sustained satisfaction in sexual and romantic partnerships.

Future research should prioritize longitudinal studies on attachment and emotion regulation to examine how attachment styles evolve over time in AYA cancer survivors and their impact on emotional regulation and relationship satisfaction. Such research could identify critical periods for targeted interventions. Our findings further highlight the need for research on how specific cancer-related complications affect relationships and on identifying vulnerable partnership stages. Such investigations could help to inform the development of targeted programs focusing on managing caregiving-related stress in long-term partnerships or enhancing communication skills in newer relationships impacted by cancer. These targeted approaches could lead to more personalized interventions that mitigate the negative impact of these factors and promote healthier relationship dynamics in survivors. Further investigation should also assess the effectiveness of interventions that actively involve partners in the therapeutic process, including the potential benefits of couple-based therapy for enhancing relationship satisfaction and sexual well-being.

By addressing these areas, future research can significantly contribute to the development of effective, nuanced, and comprehensive support systems for AYA cancer survivors, ultimately enhancing their quality of life and long-term survivorship outcomes.

## Conclusions

The identified associations between SSAT, PSAT, and attachment-related variables highlight the importance of addressing both emotional and relational dimensions in supportive care interventions tailored for AYA cancer survivors. There is a clear need for more targeted studies on attachment patterns, SSAT, and PSAT in this specific population to further refine and validate these approaches.

## Data Availability

The datasets presented in this article are not readily available because of data protection regulations concerning patient information (which assures participants that the data will not be passed on to third parties) but are available from the corresponding author upon reasonable request.
